# The Effects of Mindfulness and Buddhist Meditation Coaching on Mental Health Outcomes in College Students

**DOI:** 10.1155/2022/8178930

**Published:** 2022-11-24

**Authors:** Yuri Kim, Jaewon Khil, Wangmo-Seo, NaNa Keum

**Affiliations:** ^1^Academy of Buddhist Studies, Dongguk University, Seoul 04626, Republic of Korea; ^2^Department of Nutrition, Harvard T. H. Chan School of Public Health, Boston, MA 02138, USA; ^3^Department of Food Science and Biotechnology, Dongguk University, Seoul 04620, Republic of Korea

## Abstract

College students are vulnerable to diverse mental health disorders. We aimed to investigate whether a meditation class would be an effective means to address students' mental health challenges. Among the college students who registered for the meditation course, 256 participants were enrolled. The meditation course was a 15-week program incorporating mindfulness meditation and Ganhwa Seon (a traditional Buddhist meditation). A questionnaire was administered twice, on the first and last class of the course, collecting information on personal characteristics and six mental health indicators. A paired *t*-test was used to examine whether the meditation course conferred benefit on the mental health indicators, and logistic regression analyses were run to identify factors associated with mental health outcomes. After completing the meditation course, there were significant improvements for the adult ADHD score (*p* < 0.01) and ego identity (*p* = 0.02) but not for the other outcomes. Among college students, meditation practice may have positive effects on the adult ADHD score and ego identity.

## 1. Introduction

College students, undergoing the transition from adolescence to adulthood, are exposed to unique stressors [1]. In addition to academic pressure, college students are expected to take on adult responsibilities in financial support, personal relationships, social engagement, etc. [2]. Encountered with such stressors, many college students face mental health challenges, with the rates of mental illness rising on college campuses [2]. Thus, colleges and universities try to find ways to address the mental health crisis among students. One potential intervention may include mindfulness meditation, the most popular meditation practice in the West. Originating from Buddhist teachings, mindfulness meditation is a mental training framework to cultivate awareness of what one is sensing and feeling in the moment through nonjudgmental observation [3].

A previous study comprising a systematic review of randomized controlled trials indicated that mindfulness meditation could be associated with the regulation of the immune system, for instance, the decrease in proinflammatory processes or the elevation in cell-mediated defense systems [3]. Though there is a certain level of difference depending on the level of trait mindfulness, another study of college students showed that the anxiety state decreased as the mindfulness state increased and that was an improvement in attention and executive function performance [4]. Also, a separate study conducted with 152 Taiwanese university freshmen over the course of a semester-long mindfulness meditation course found improvements in learning effectiveness as well as the cognitive performance of attention and memory [5].

Of note, referred to as Ganhwa Seon, it represents the mainstream of meditation within Korean Buddhism [6]. Ganhwa Seon involves raising a specific question, termed “hwadu,” and directing one's full attention to the question in daily life to get deeply absorbed in the single-mindedness of questioning [6]. While mindfulness meditation is widely accepted by people regardless of one's religion and culture, as well as transnationally, the practice of Ganhwa Seon is often limited to Buddhists in Korea. At Dongguk University in Korea, as a Buddhism-based university, students are required to take a meditation course, in which Buddhist spiritual leaders guide students to practice mindfulness meditation and Ganhwa Seon. In this study, among college students, we aim to investigate whether meditation practice performed as class activities affects six mental health outcomes. (1) Adult attention-deficit hyperactivity disorder (ADHD): inattention, hyperactive-impulsive symptoms, or their combinations [7]; (2) ego identity: the sense of self to experience who they are [8]; (3) perceived stress: the extent to which individuals perceive their life circumstances to be stressful, unpredictable, and uncontrollable [9]; (4) self-efficacy: individual confidence in the ability to take required actions to fulfill specified objectives [10]; (5) self-esteem: diverse and intricate mental states related to self-perception[11]; and (6) spiritual well-being: the intrinsic sense of contentment [12].

## 2. Materials and Methods

### 2.1. Study Participants

**Figure 1:**
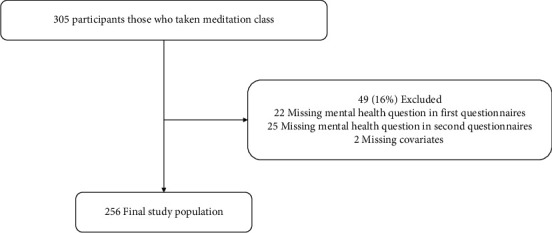
The flow chart of the study population formation.

We recruited students who registered for a meditation course at Dongguk University in Seoul, Korea, in 2018. Initially, 305 subjects were recruited for the study participants. We excluded individuals with missing information on the first questionnaire (*n* = 22), the second questionnaire (*n* = 25), and/or covariables (*n* = 2). After these 16% exclusions, the final analytic sample included 256 participants (133 males and 123 females). Missing data were defined as the absence of responses to at least one of the mental health questionnaires (Figure 1).

The researcher gave participants a full explanation of the research purpose before enrollment, and consent to participate was obtained voluntarily. This study was approved by the Dongguk Institutional Review Board (IRB number: DUIRB-201810-13).

### 2.2. Meditation Course

As a Buddhism-based institution, Dongguk University sets successful completion of the meditation course as a graduation requirement. Instructed by a Buddhist spiritual leader with a Ph.D. in the field of Zen, the course aims to introduce students to mindfulness meditation and Ganhwa Seon and motivate them to practice meditation in their daily lives. The course comprised 15 weekly, 50-minute meditation sessions and was graded on a pass/fail system. The class lectures encompassed the following plan: (1) instructor introductions; (2) meditation etiquette; (3) why modern individuals require meditation; (4) achieving dreams through meditation; (5) varied methods of meditation; (6) Ganhwa Seon 1; (7) mindfulness meditation (imagery meditation); (8) watching a meditation video; (9) mindfulness meditation (meditation with music); (10) mindfulness meditation (meditation with sound); (11) mindfulness meditation (conversation meditation); (12) mindfulness meditation (meditation for happiness); (13) mindfulness meditation (meditation through speech); (14) Ganhwa Seon 2; (15) meditation as a lifestyle.

### 2.3. Data Collection

Participants responded to a questionnaire twice during the first and last classes of the meditation course. The questionnaire collected information about personal information (e.g., age, sex), grade (i.e., students' academic year at the time of course registration), major, adjusting to college life, socioeconomic status, religion, knowledge of Buddhism, the importance of religion in life, and six mental health indicators (adult ADHD, ego identity, perceived stress, self-efficacy, self-esteem, and spiritual well-being).

The adult ADHD score was assessed by the Korean version of the Conners adult ADHD rating scale-26 items, a 26-item instrument developed to measure the presence and severity of ADHD symptoms in adults [13]. Each item was rated on a 4-point scale from 1 (never) to 4 (almost always), and the composite score can range from 26 to 104, with higher scores indicating higher levels of ADHD.

Ego identity was assessed by the ego-identity scale instrument, which was developed in Korea to measure various aspects of ego identity [14]. The scale consisting of 48 items, covers areas of independence, self-acceptance, future certainty, goal orientation, proactive personality, and intimacy. Each item was rated on a 5-point scale from 1 (never) to 5 (almost always), and the composite score can range from 48 to 240, with higher scores indicating higher levels of ego identity.

Perceived stress was measured by the Korean version of the perceived stress scale, a 10-item instrument developed to assess the degree to which situations in one's life are appraised as stressful [15]. With each item rated on a 5-point scale from 1 (never) to 5 (almost always), the composite score can range from 10 to 50, and higher scores indicate higher perceived stress.

Self-efficacy was measured by the new general self-efficacy scale, an 8-item instrument designed to measure the degree of a person's belief in one's ability to achieve one's goal despite difficulties [16]. With each item rated on a 5-point scale from 1 (never) to 5 (almost always), the composite score can range from 8 to 40, and higher scores indicate higher self-efficacy.

Self-esteem was measured by the Korean version of the Rosenberg self-esteem scale, a 10-item instrument designed to assess positive and negative feelings about the self [17]. With each item rated on a 4-point scale from 1 (never) to 4 (almost always), the composite score can range from 10 to 40, and higher scores reflect higher levels of self-esteem.

Spiritual well-being was measured by the Functional Assessment of Chronic Illness Therapy—Spiritual Well-Being, a 12-item questionnaire designed to measure spiritual well-being in people with cancer and other chronic illnesses [18]. With each item rated on a 5-point scale from 1 (never) to 5 (almost always), the composite score can range from 12 to 60, and higher scores reflect higher well-being.

### 2.4. Statistical Analysis

To understand the characteristics of the study population before the start of meditation classes (i.e., at baseline), we calculated proportions for categorical variables and mean and standard deviation for continuous variables.

To evaluate whether mental health outcomes (adult ADHD, ego identity, perceived stress, self-efficacy, self-esteem, and spiritual well-being) changed significantly after meditation classes in a one-grouppretest-posttest design, we used the paired *t*-test. A parametric test was chosen based on the central limit theorem, which states that the sampling distribution of differences approximates a normal distribution when the sample size is above 30 [19]. In general, individuals respond differentially to meditation classes. By defining change in outcome scores as a binary variable (1: improvement in scores; 0: no improvement in scores), we performed a multivariable logistic regression analysis to calculate the odd ratios (ORs) of the improvement *P*_case_/(1 − *P*_case_) ∕*P*_control_/(1 − *P*_control_)*∗*P=probabilities of event occurrence to identify factors associated with improvements in spiritual and mental health outcomes. The levels of the outcome variables were defined as 1 for individuals who showed improvement after meditation classes and 0 for those who did not show any improvement. The models were adjusted for the baseline characteristics (i.e., sex, grade, major, college adaptation, socioeconomic status, religion, degree of knowledge of Buddhism, degree of importance of religion in life, and instructors) as well as the level of the outcome before the start of the medication class.

*p* value less than 0.05 is considered statistically significant. All statistical analyses were performed using SAS 9.4 (SAS Institute, Cary, NC, USA).

While the final sample was not determined based on a priori power calculation, based on a post-hoc power calculation setting *α* error rate at 0.05, our study has 89% power to detect a mean difference of 1 in the outcome score (standard deviation of the difference: 5).

## 3. Results

The characteristics of the 256 participants are presented in Table 1. The study population was almost equally divided by gender. Approximately 46% and 30% of participants were freshmen and sophomores, respectively. The number of students in the College of Science was the highest, and the number of students in the College of Humanities and Social Sciences was the next. About 12% of participants were Buddhists, and 88% were Christians, believers of other religions, or atheists. Most of the participants had little or fair knowledge about Buddhism, and about 41% of students answered that religion had no effect on their lives.

**Figure 2:**
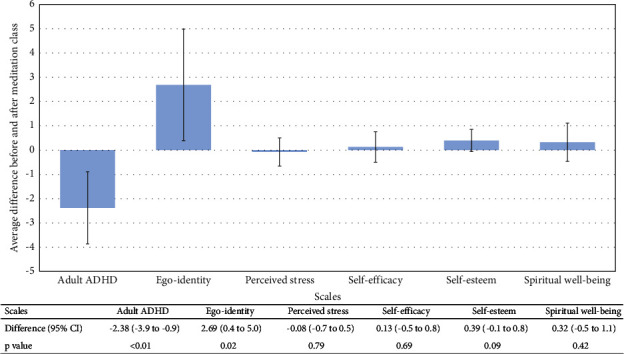
Average differences in spiritual and mental health outcomes before and after meditation class.

The meditation classes conferred varied benefits according to mental health outcomes. For the adult ADHD score and ego identity, significant improvements were observed after the meditation class. (Changing in adult ADHD score −2.38, *p* < 0.01; ego identity 2.69, *p* = 0.02). Perceived stress, self-efficacy, self-esteem, and spiritual well-being improved modestly, but the changes were not statistically significant. (Perceived stress −0.08, *p* = 0.79; self-efficacy 0.13, *p* = 0.69; self-esteem 0.39, *p* = 0.09; and spiritual well-being 0.32, *p* = 0.42) (Figure 2).

Individuals benefited from the meditation class differentially with regard to adult ADHD score change and ego identity (Table 2). For a change in adult ADHD score, students were more likely to improve their adult ADHD score after the meditation class when they had a higher ADHD score at baseline (OR 1.05, 95% CI 1.03–1.08, *p* < 0.01), in college of education (OR 3.58, 95% CI 1.25–10.23, *p* = 0.03), and had a better adaptation to college life (OR 2.04, 95% CI 1.05–4.00, *p* = 0.04) at baseline. For ego identity, students were associated with an increased ego identity after the meditation class when they had a lower ego identity at baseline (OR 0.98, 95% CI 0.96–0.99, *p* < 0.01), male sex (OR 3.04; 95% CI 1.66–5.59, *p* < 0.01), in college of education (OR 5.48, 95% CI 1.70–17.63, *p* < 0.01), and of Buddhist (OR 2.58, 95% CI 1.00–6.61, *p* = 0.05) at baseline.

## 4. Discussion

In this study of 250 university students, we investigated the effect of practicing mindfulness meditation and Ganhwa Seon as class activities on six mental health outcomes (adult ADHD, ego identity, perceived stress, self-efficacy, self-esteem, and spiritual well-being).

We observed that in-class meditation practice conferred statistically significant improvements in adult ADHD scores and ego identity. Notably, the beneficial effects of the meditation class on these two outcomes varied by several baseline characteristics. For a change in adult ADHD score, students were more likely to benefit from the meditation class when they had a severe adult ADHD score, were from a college of education, and were better adapting to college life; for ego identity, students were more likely to benefit when they had a lower ego identity, were of the male sex, were from a college of education, and were Buddhists. Upon completing the meditation course, perceived stress, self-efficacy, self-esteem, and spiritual well-being changed positively, but the improvements were not statistically significant.

Adult ADHD is a psychiatric condition suggested to be caused by a complex combination of genetic and environmental risk factors [20]. Its major symptoms include difficulties with initiating work, self-organization, and prioritization; varying levels of attention to details; and poor persistence in work requiring continuous mental efforts [21]. Consistent with our findings, previous studies have shown that mindfulness meditation improves adult ADHD scores. In a study of 24 adults and 8 adolescents in the US, an 8-week mindfulness training program resulted in improvements in self-reported ADHD symptoms [22]. In another study conducted among Taiwanese university students, students who took a one-semester mindfulness meditation course showed improvements in cognitive attention and memory compared to control students who did not take the meditation course [5]. According to neuroimaging studies, the anterior cingulate cortex has been implicated in several cognitive functions, including attention [23, 24], and meditation practice was shown to activate the anterior cingulate cortex practice, contributing to attention maintenance [24, 25]. In addition, cortical thickness, whose reduction is a marker of adult ADHD [26], was higher in the dorsal anterior cingulate cortex among experienced meditators compared with control subjects in anterior cingulate cortex magnetic resonance neuroimaging studies [27].

Ego identity refers to the sense of self that enables individuals to experience a sense of who they are [8]. Previous studies have shown that regular engagement in meditation improved self-knowledge, facilitating individuals to become fully attentive to what is happening without any judgements [28]. In addition, mindful meditation was associated with increased self-acceptance, which in turn helps a foster healthy sense of one's true self [29]. While no previous studies investigated the direct effect of Ganhwa Seon on ego identity, one of the most common questions that is consistently asked during the practice is “who am I” [6], which relates to the pursuit of ego identity. The beneficial effects of meditation on ego identity are also supported by biological mechanisms. The prefrontal cortex plays a critical role in higher brain functions, including personality formation [30]. Neuroimaging studies suggested that enhanced activation of the ventromedial prefrontal cortex might promote the development of identity [31]. In a study of healthy older adults, participants who engaged in eight-week meditation training showed enlargements in a prefrontal network involving the ventromedial prefrontal cortex compared with those engaged in control relaxation training [32].

In our study, not all students benefitted equally from the meditation class. For both adult ADHD score change and ego identity, students with worse outcome conditions (i.e., higher adult ADHD score, lower ego identity) at baseline and from the college of education experienced greater improvements after meditation practice, compared with students in their counterparts. Much as nutritional supplements are more likely to benefit individuals with nutrient deficiencies than individuals who are nutritionally replete [33], meditation practice may be more effective among individuals experiencing mental health problems and, thus, have more room for improvement than among healthy individuals. In the case of students from the College of Education, due to their major, they tend to have a greater appreciation for education. This mindset and attitude might have motivated these students to exert greater efforts during the meditation class, which in turn led to the greater benefit of meditation.

Specifically, for change in adult ADHD scores, students who reported being well-adjusted to college life were more likely to improve their adult ADHD score after the meditation practice than students who did not. Academic satisfaction is an important determinant of college adjustment, which predicts achievement [34]. In light of this, students with better adjustment to college life might have actively participated in the meditation class and thus benefitted from it to a greater degree.

In raising the level of ego identity, the meditation class was more effective among male students and Buddhists than among female students and students believing in other religions. As mentioned previously, a mechanism by which meditation promotes the development of ego identity is through the activation of the ventromedial prefrontal cortex [31]. Evidence suggests that men exhibited stronger connectivity in the prefrontal cortex regions [30], which may make men more sensitive to the stimulating effect of meditation for the promotion of ego identity. Regarding the greater benefit of meditation for Buddhists, the inclusion of Ganhwa Seon, a traditional Buddhist meditation that strives to find the answer to a question of choice, in the class might be a relevant factor. In Korea, meditation, in general, is regarded as a part of Buddhist practice, and the meditation class guided by Buddhist spiritual leaders might have created an unfamiliar environment that hindered non-Buddhists from immersing themselves in meditation practice. Conversely, Buddhist students, out of reverence for Buddhism and Buddhist spiritual leaders, might have engaged in meditation practice more fully and truly rather than approaching meditation functionally or technically, and thus might have reaped the benefit [35]. Our finding is also aligned with the observation that individuals who grow up in a Buddhist culture have a tendency toward higher levels of ego development [28].

Past studies have shown the beneficial effects of meditation on improving perceived stress, self-efficacy, self-esteem, and spiritual well-being, but in our study, the meditation class did not confer significant benefits for these outcomes [36–39]. Differences in the type and setting of meditation might have contributed to the inconsistent findings. Unlike the previous studies [36–39] that examined the effect of mindful meditation, the most widely practiced meditation and also employed as a part of psychotherapy in clinical settings [40], our study evaluated an integrated form of mindfulness meditation and Ganhwa Seon. Ganhwa Seon might have enriched the medication class by introducing students to a different variation of meditation than mindfulness meditation alone. However, with Ganhwa Seon rooted in Buddhism and led by Buddhist spiritual leaders, students could have misinterpreted Ganhwa Seon as a religious intervention rather than a neutral meditation practice, which might have created some disapproval and dislike of the meditation practice, leading to no efficacy. In fact, a study found that college students had different preferences for different meditation methods [41]; and thus, choosing the right type of meditation for oneself appears critical to reaping the benefits of meditation practice.

There are several strengths in our study. This is the first study to investigate the combined effects of the two representative meditations, mindful meditation (the most popular meditation practice in the West) and Ganhwa Seon (the mainstream meditation within Korean Buddhism). By including about 250 participants, it is a large-scale study compared to other previous studies on meditation, which are often limited to less than 100 [3]. The large study size allowed for analyses to explore characteristics that can help identify individuals who would benefit most from meditation practices.

Our study also has several limitations. First, because students in the meditation class were graded on a pass/fail basis rather than with letter grades, they might not have fully engaged in the meditation practice. This might have led to an underestimation of meditation's benefits. Second, the medication course was offered by different instructors, and thus, different teaching styles across instructors might have created differences in the meditation effect even though the course syllabus was identical across classes. However, spiritual leaders completed the same course at the same university to lead the course, and there are unified guidelines in the textbook for running the course. Furthermore, in our multivariable regression analysis, we adjusted for an instructor. Thus, our effect size measures the effect of meditation, removing variation due to differences in instructors. Third, the unique characteristics of our study setting, in which Buddhist spiritual leaders guided meditation incorporating Buddhism and students were forced to engage in meditation as part of a required course rather than opting to practice meditation, might have created emotional resistance from students of other religions, which might have underestimated the benefit of meditation. Last, the meditation in our study was conducted in a class held at a Buddhist university, and only those who answered the questionnaires twice were included in the study. In this regard, the subjects of our study are relatively familiar with meditation, so their interest in meditation may be different from that of the general population. Due to this, the effect of meditation we found may be overestimated or underestimated compared to the general population.

## 5. Conclusions

A semester meditation course incorporating mindfulness meditation and Ganhwa Seon was associated with improvements in adult ADHD scores and ego identity among college students. The beneficial effects of meditation courses varied according to several baseline characteristics, which provide evidence for targeted meditation practice. For effective control of mental health issues among college students, future studies are warranted to find characteristics that can identify a subgroup of individuals who would benefit most from meditation practice.

## Figures and Tables

**Table 1:** Descriptive characteristics of the study population at baseline.

Individual characteristics	
Sex, *n* (%)	
Men	133 (52.2)
Women	123 (47.8)
Grade, *n* (%)	
Freshmen	117 (45.7)
Sophomore	76 (29.7)
Junior	39 (15.2)
Senior	24 (9.4)
Major, *n* (%)	
College of humanities and social sciences	99 (38.7)
College of science	100 (39.1)
College of arts and sports, others	31 (12.1)
College of education	26 (10.2)
Adjusting to college life, *n* (%)	
Good	122 (47.7)
Poor	134 (52.3)
Household income, quantile, *n* (%)	
First (highest)	106 (41.4)
Second	108 (42.2)
Third (lowest)	42 (16.4)
Religion, *n* (%)	
Atheism or others	159 (62.1)
Buddhism	30 (11.7)
Catholic	21 (8.2)
Protestantism	46 (18.0)
Knowledge of Buddhism, *n* (%)	
No knowledge	16 (6.3)
Little knowledge	80 (31.3)
Fair knowledge	147 (57.4)
Well knowledge	13 (5.1)
Importance of religion in life, *n* (%)	
Not important	106 (41.4)
Not so important	62 (24.2)
Somewhat important	50 (19.6)
Very important	38 (14.8)
Before meditation class	
Adult ADHD level, mean (sd)	64.6 (15.0)
Ego identity level, mean (sd)	163.7 (27.0)
Perceived stress level, mean (sd)	27.5 (6.0)
Self-efficacy level, mean (sd)	31.3 (5.8)
Self-esteem level, mean (sd)	35.3 (5.1)
Spiritual well-being level, mean (sd)	41.0 (7.8)
After meditation class	
Adult ADHD level, mean (sd)	62.3 (16.6)
Ego identity level, mean (sd)	166.4 (29.0)
Perceived stress level, mean (sd)	27.5 (6.3)
Self-efficacy level, mean (sd)	31.5 (5.3)
Self-esteem level, mean (sd)	35.6 (5.2)
Spiritual well-being level, mean (sd)	41.3 (7.8)

Abbreviations: sd, standard deviation; *n*, number.

**Table 2:** Odds ratios and 95% CIs for the associations between baseline characteristics and improvements in ego identity and adult ADHD levels after meditation classes.

Individual characteristics	Adult ADHD		Ego identity	
	Multivariate adjusted OR	*p* value	Multivariate adjusted OR	*p* value
Baseline level before meditation class	1.05 (1.03–1.08)	<0.01	0.98 (0.96–0.99)	<0.01
Sex				
Men	1.12 (0.64–1.97)	0.69	3.04 (1.66–5.59)	<0.01
Women	Ref		Ref	
Grade				
Freshmen	Ref		Ref	
Sophomore	0.61 (0.33–1.15)	0.17	1.06 (0.55–2.03)	0.53
Junior	1.44 (0.64–3.20)	0.09	2.13 (0.93–4.89)	0.08
Senior	0.61 (0.23–1.61)	0.34	1.05 (0.39–2.87)	0.65
Major				
College of humanities and social sciences	Ref		Ref	
College of science	1.30 (0.71–2.38)	0.47	0.63 (0.34–1.17)	0.17
College of arts and sports, others	1.19 (0.50–2.81)	0.43	2.10 (0.83–5.36)	0.48
College of education	3.58 (1.25–10.23)	0.03	5.48 (1.70–17.63)	<0.01
Adjusting to college life				
Good	2.04 (1.05–4.00)	0.04	1.59 (0.82–3.09)	0.17
Poor	Ref		Ref	
Socioeconomic status				
First (highest)	1.62 (0.73–3.58)	0.56	1.78 (0.78–4.05)	0.21
Second	1.86 (0.82–4.20)	0.21	1.47 (0.63–3.42)	0.75
Third (lowest)	Ref		Ref	
Religion				
Protestantism, Catholic, atheism or others	Ref		Ref	
Buddhism	2.05 (0.82–4.20)	0.13	2.58 (1.00–6.61)	0.05
Knowledge of Buddhism	1.02 (0.68–1.55)	0.91	0.73 (0.47–1.11)	0.14
Importance of religion in life	0.98 (0.75–1.27)	0.85	0.95 (0.73–1.25)	0.73

## Data Availability

The data that support the findings of this study are available from the corresponding author upon reasonable request.
