# Correction to “The Effects of Mindfulness and Buddhist Meditation Coaching on Mental Health Outcomes in College Students”

**DOI:** 10.1155/ecam/9847542

**Published:** 2025-10-03

Y. Kim, J. Khil, Wangmo-Seo, and N. Keum, “The Effects of Mindfulness and Buddhist Meditation Coaching on Mental Health Outcomes in College Students,” *Evidence-Based Complementary and Alternative Medicine* 2022 (2022): 8178930, https://doi.org/10.1155/2022/8178930.

In the article titled “The Effects of Mindfulness and Buddhist Meditation Coaching on Mental Health Outcomes in College Students,” there was error in Reference 21. The correct Reference 21 is shown below:

J. Biederman, “Attention-Deficit/Hyperactivity Disorder: A Life-Span Perspective,” *Journal of clinical psychiatry* 59, no. 7 (1998): 4–16.

We apologize for this error.

